# Cases from Practice

**Published:** 1894-05

**Authors:** 

**Affiliations:** Kiel


### CASES FROM PRACTICE.

By Dr. Kunkel, Kiel.

[From *Archiv. für Homœopathie*, translated by A. McNeil.]

Miss E., æt. fifteen, consulted me October 26th, 1892. She has suffered for two years, with longer or shorter intervals, from epileptic spasms. I could obtain no satisfactory description of the form which they assumed. She said that at first they appeared in the daytime, but now they come at night. Functions tolerably normal, only before her menses, which were irregular, quite violent pelvic pains. After waking in the morning still sleepy. Sleep unrefreshing. Very great tendency to sweat, particularly on the back and in the axillæ. She avoids sitting for any length of time, and prefers to "walk about." After paroxysm headache, with vomiting. Sepia$^{cc}$, a dose every seventh evening.

December 7th.—Has not had an attack. Menses appeared without pain. Her general health is also better. The sleepiness in the morning has disappeared entirely. Sepia$^{cc}$, a dose every ninth evening. (Why continue the medicine even with a lengthened interval ?—Trans.)

January 3d, 1893.—Had three paroxysms. December 24th, with the last one her face was quite blue. Green vomiting after each. Since then quite well. Sepia$^{cc}$, a dose every fourteenth day.

February 13th.—Her health is good all the time. Menses painless. Drawing in the tips of the fingers on waking in the morning. Sepia$^{300}$. (Prepared under my own supervision from the 200); a dose every fourteen days.

May 2d.—Has not had an attack. No complaints of any kind. No medicine.

Up to the middle of July has not had a return.

As to the hope of a radical cure, that is the more to be expected from the occurrence of three paroxysms in one day followed immediately by good health. I have observed in several such cases that the last epileptic attack was the severest, a fact which is not surprising when we consider that the paroxysm itself does not represent the disease, but in both cases signifies an unloading of the diseased organism. In intermittent fever, likewise, an unusually violent attack announces a radical cure, more especially in old cases or in those that have been treated by different allopathic drugs.

### Ischias Sciatica (Neuralgia Ischiatica).

Mrs. S., æt. forty-seven, had suffered several years ago from an eruption on different parts of the body. She consulted me

August 3d, 1892, on account of an ischias on the right side, from which she has suffered since last February. She says that she was at first sick for eight days, during which the pains were on both sides. Then it settled in her right hip, and then went downwards, and is now so that she suffers all the time. She cannot endure sitting or keeping quiet long at a time. Aggravation at night in bed; she must lie on the painful side. She sweats profusely. Before this disease set in she suffered from headache on awaking in the morning. This has now disappeared. General health good. Sepia$^{40}$ [prepared by myself], a dose every seventh evening.

September 8th.—The primary action of the medicine was violent, and it continued a week. She perceived a clear improvement in every direction after the second dose, including sleep. I now gave the same remedy every seventh evening, and no other was required to complete the cure.

I found no remedy so often indicated as Sepia in the treatment of ischias. It has as characteristics aggravation when sitting, when rising from a seat, after moving, lying on the painless side, and sometimes by great exertion, while in other cases the pain decreases in inverse ratio to the increased exertion.

Causticum, which also deserves consideration in ischias, has aggravation in direct proportion to the bodily exertion involved. However, a comparatively short rest relieves the pains, and the paralyzed sensation and the gone-to-sleep sensation which characterize it.

### Ischias.

K., a countryman, always well until the present illness, consulted me July 25th, 1891. He has suffered since last January from ischias of the left hip. He feels no pain when working in an upright position, but it appears immediately on stooping. Slight sweat of the entire body, particularly of the head. Sultry air is intolerable. When sitting long the pain is constant, and disappears on rising and walking about. Free from pain at night. Aggravation on catching cold. Sepia$^{30}$, a dose every seventh evening.

September 5th he is almost well, except that on stooping there is some pain below the left trochanter. Formerly it was in the whole extremity, which soon disappeared on motion. Aggravation in the warmth of the bed, as in the case before the last. Sepia cures but rarely. When an aggravation occurs at night it is on account of the quietness.

### Chorea.

The thirteen-year-old daughter of a countryman consulted me on the 7th of April, 1889. She has suffered from St. Vitus's dance for a year. It arose from fright, it is believed. It was first observed in school that she made all kinds of movements with her mouth, right hand, and right foot. They increased gradually so that she cannot write well. Her thinking capacity is becoming gradually weaker. She has no pain. Her functions are in a measure normally performed, only her disposition is many times somewhat sad. When sleeping she lies constantly on the left side. Speech is constantly becoming more impeded; frequent muscular twitching. Causticum$^{30}$, a dose every seventh evening. Before the sixth dose was taken she was fully restored.

